# On Application of Lightweight Models for Rice Variety Classification and Their Potential in Edge Computing

**DOI:** 10.3390/foods12213993

**Published:** 2023-10-31

**Authors:** Muhammad Javed Iqbal, Muhammad Aasem, Iftikhar Ahmad, Madini O. Alassafi, Sheikh Tahir Bakhsh, Neelum Noreen, Ahmed Alhomoud

**Affiliations:** 1 Department of Computer Science, University of Engineering and Technology Taxila, Taxila 47050, Pakistan; javed.iqbal@uettaxila.edu.pk (M.J.I.); muhammadaasem@gmail.com (M.A.); 2 Department of Information Technology, Faculty of Computing and Information Technology, King Abdulaziz University, Jeddah 21589, Saudi Arabia; 3 Department of Computer Science, Cardiff Metropolitan University, Cardiff CF5 2YB, UK; sbakhsh@cardiffmet.ac.uk; 4 Department of Computer and Information Sciences, Gulf Colleges, Hafr Al Batin 39952, Saudi Arabia; 5 Department of Computer Sciences, Faculty of Computing and Information Technology, Northern Border University, Rafha 91911, Saudi Arabia

**Keywords:** rice varieties, rice types, deep learning, end-to-end learning, transfer learning

## Abstract

Rice is one of the fundamental food items that comes in many varieties with their associated benefits. It can be sub-categorized based on its visual features like texture, color, and shape. Using these features, the automatic classification of rice varieties has been studied using various machine learning approaches for marketing and industrial use. Due to the outstanding performance of deep learning, several models have been proposed to assist in vision tasks like classification and detection. Regardless of their best results on accuracy metrics, they have been observed as overly excessive for computational resources and expert supervision. To address these challenges, this paper proposes three deep learning models that offer similar performance with 10% lighter computational overhead in comparison to existing best models. Moreover, they have been trained for end-to-end flow to demonstrate minimum expert supervision for pre-processing and feature engineering sub-tasks. The results can be observed as promising for classifying rice among five varieties, namely Arborio, Basmati, Ipsala, Jasmine, and Karacadag. The process and performance of the trained models can be extended for edge and mobile devices for field-specific tasks autonomously.

## 1. Introduction

The classification of rice varieties is a critical and complex task that has significant implications for agriculture, trade, and food quality. Traditional approaches often depend on human expertise, which is subjective and not easily scalable. Artificial intelligence, particularly deep learning, offers new possibilities for automating this task by learning features directly from data. In this broader context, it is noteworthy that rice is among the oldest cereal grains and continues to serve as a staple food for nearly 50% of the world’s population [1,2]. Beyond its nutritional value, the classification and quality of rice varieties play a crucial role in determining market prices and ensuring food safety. Generally, there are various aspects that establish price value for rice including color, texture, shape, etc. [3,4]. Each rice type has a separate texture, taste, and other distinct properties that are known for different applications. Human experts can distinguish rice just by naked-eye visual inspection. Such expertise is in demand in trade markets, restaurants, and the agriculture industry all the time. This increases the need for discovering other means for such expertise. In the past decades, artificial intelligence has gained popularity in mimicking human intellectual tasks like image analysis, classification, and localization. 

Deep learning is a de-facto sub-field of artificial intelligence that learns from examples and creates skill as a model that can be used to predict new or unseen instances [5]. It has achieved human expert-level performance in medical vision tasks [6,7,8] and is gaining popularity in agriculture [9,10,11,12,13,14,15,16]. Parallel to deep learning, a trend of tasks in the agricultural domain can also be observed in classical machine learning approaches. Such approaches are generally less complex than deep learning models but are limited in their ability to learn features from data automatically [17]. They require proper pre-processing analytical and feature engineering tasks [18]. Deep learning, on the other hand, uses neural networks with multiple layers to automatically learn features from data [19]. These neural networks can learn hierarchical representations of data, which allows them to achieve better performance on tasks such as image and speech recognition. They are mostly more complex than traditional machine learning models and require larger amounts of data and computational power to train [17]. The aim of this paper is to overcome the dependency on pre-processing tasks while maintaining the highest performance in lightweight computational overhead.

The proposed approach in this paper aims to address a critical issue in agriculture: the accurate and efficient classification of rice varieties. The suitability of the approach lies in several key factors like Computational Efficiency, Accuracy, Flexibility and Adaptability, and Practical Utility. The proposed approach is suitable because it balances computational efficiency with high accuracy while providing the flexibility to adapt to new challenges. It also takes into consideration the practical aspects of deployment, making it a well-rounded solution to the critical problem of rice variety classification. 

The key findings of our research indicate that the proposed models achieve high classification accuracy across rice varieties. This performance is further highlighted by the models reaching their peak accuracy efficiently. The employment of Keras callbacks such as EarlyStopping and ReduceLROnPlateau optimized the training process, further contributing to the models’ robustness and reliability. On the advantages front, our models stand out for their low computational cost and high efficiency, making them ideally suited for real-time inference in edge computing scenarios. The architecture’s inherent flexibility allows for fine-tuning to accommodate new rice varieties, thus providing a dynamic and scalable solution. Furthermore, the practical utility of our models is evident from their potential for integration into mobile applications, offering a real-time, on-the-field classification tool that is both robust and reliable.

This paper makes several key contributions to the field of rice variety classification using deep learning:

- Lightweight Models: We introduce lightweight deep learning models that maintain high classification accuracy while reducing computational overhead, addressing the challenge of computational complexity in existing models.
- Reduced Pre-processing: Our models are designed to minimize the need for expert-level pre-processing and feature engineering, making the system more accessible for non-specialists.
- Balance of Performance and Efficiency: Unlike existing studies that focus predominantly on performance metrics, this paper aims to strike a balance between performance and computational efficiency.
- Parameter Uncertainty: We include an in-depth analysis of model complexity and parameter uncertainty, aspects often overlooked in the existing literature.
- Real-world Applicability: Our approach is particularly useful for resource-constrained settings, broadening the scope of automated rice classification techniques.

The remainder of this paper comprises four sections. The subsequent section provides a concise summary of pertinent literature. Section 3 delves into the methodology employed in the proposed study. In Section 4, outcomes are articulated and the enhancements resulting from the proposed work are examined. The paper concludes with a discussion of implications and avenues for future research.

## 2. Literature Review

Deep learning has demonstrated great potential in agriculture to assist in various tasks [20]. This includes the classification of plant diseases, pest identification, and crop-type classification. Similar attempts offer improvements in crop yields, reductions in resource usage, and an increase in efficiency in the agricultural industry. However, deep learning models with many layers bear much larger computational costs and complexity compared to traditional machine learning models. 

In [9], the authors proposed a fruit-counting pipeline using deep learning. The pipeline consists of a blob detector and counting algorithm. Both of the tasks use convolutional neural networks. A similar approach is described in [10] that employs deep learning architecture for crop-type classification using multitemporal multisource satellite imagery. This architecture uses deep learning for optical imagery segmentation, and an ensemble of supervised neural networks. 

To reduce the computational cost, some custom models have also been proposed in the literature. Lighter models have been discussed in [11] for disease detection. For the classification of maize leaf disease, GoogLeNet and Cifar10 were considered in [12]. In comparison to Visual Geometry Group (VGG) and AlexNet models, their considered models perform better with less computational complexity. A multilayer convolutional neural network has been proposed for the diagnosis of anthracnose disease in mango leaves in [13]. Likewise, a convolutional neural networks-based model has been proposed in [14] to detect apple leaf diseases in real-time. This model consists of GoogLeNet Inception and Rainbow concatenation.

M. Koklu et al. [21] conducted experiments on a dataset using three models: Artificial Neural Network (ANN), Deep Neural Network (DNN), and Convolutional Neural Network (CNN). The ANN and DNN share the same architecture for the input and output layers. They take 106 features as input and produce an output of 5 neurons. ANN consists of only one hidden layer that comprises 100 neurons while DNN contains two layers each comprising 50 neurons. VGG-16 has been selected as a CNN model that has been tailored to 5 neurons output. The performances of ANN, DNN, and CNN have been reported as 99.87%, 99.95%, and 99.99%, respectively.

The reviewed literature mostly focuses on achieving high performance. This increases the need to highlight and address other major challenges including higher computational cost and dependency on skillful experts for pre-processing sub-tasks. For instance, rice classification has been proposed in [22] by using Logistic Regression, Multilayer Perceptron, about 4,550,000 results (0.40 s) Support Vector Machine (SVM), Decision Tree, Random Forest, and k-Nearest Neighbor. Although this achieved the best performance, it required expert efforts for pre-processing and morphological Feature Extraction before classification. Similar studies can be found in [23,24,25,26,27,28] where classifiers were trained after feature extraction which were mostly based on color, shape, and/or morphology. In the same era, deep learning architectures have also been proposed for grain classification [21,26,29,30,31,32,33,34]. The employed deep learning approaches used in these studies were quite deep and large. On one hand, such deeper architectures offer flexibility for generalization but demand higher memory and computational resources. The CNN-based architectures mostly need a Graphics Processing Unit (GPU) requirement for training and for inferences when quick results are expected. Table 1 summarizes the research findings, challenges, and contributions in this area.

## 3. Materials and Methods

The aim of this research is to optimize the process and product so that limited-resource devices can adapt it in agile methodology. To address the computational challenges associated with deep learning models, this paper proposes a novel approach using lightweight architectures. Our solution aims to provide the same level of accuracy in rice variety classification as traditional deep learning models, but with significantly reduced computational overhead. This makes it particularly suited for real-world applications where computational resources may be limited, thereby broadening the scope and accessibility of automated rice classification techniques. The traditional process for machine learning solutions is time consuming due to several factors. Human experts examine the datasets and create a pipeline for pre-processing. Feature analysis, selection, and/or dimensionality reduction are most noticeable in the pipeline. Next, several filters are applied and tested before and during feature extraction. After feature extraction, classification algorithms are applied and selected based on defined evaluation matrices. Once the model is finalized, a software application/app is developed that incorporates the custom pipeline of pre-processing, feature extraction, and classification. The main drawback to this approach is the demanding time and expertise that are in direct proportion to the cost as well. Our approach is a depiction of agile software development where new changes are welcomed all the time and delivered at a faster pace. 

### 3.1. Dataset

**Figure 1:**
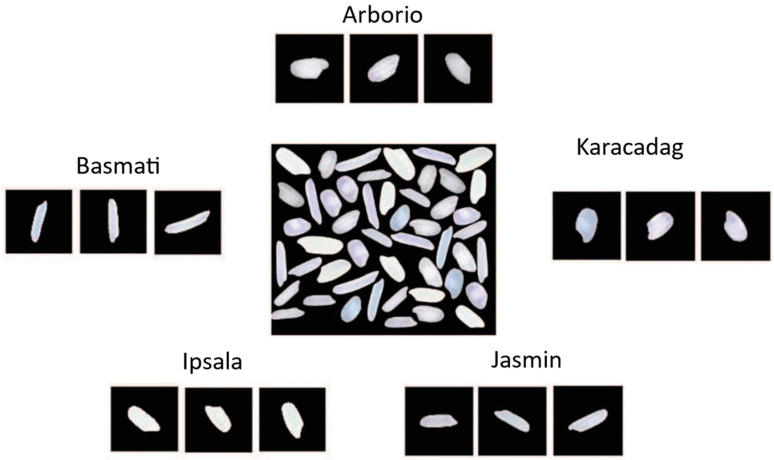
Five classes of rice from the dataset.

The dataset for this research is publicly available and has been taken from the Kaggle platform. It contains five various types of rice (see Figure 1) organized in separate directories as per each class name, i.e., Arborio, Ipsala, Basmati, Karacadag, and Jasmine. The total number of images in the dataset is 75,000 that has been equally distributed to each class of 15,000 images. All images are in RGB format with a 250 × 250 pixels size resolution.

### 3.2. Mobile Optimum Models 

Mobile phone devices are getting better in terms of processing and storage resources. Furthermore, app development frameworks like android studio have promoted a rapid application development strategy. This creates an opportunity for field workers to perform digitization activities right in the field. Two aspects are, however, still challenging for such a work environment. First is battery performance and second is internet coverage. This urges the need for optimal computations that are performed using edge devices without the need to access an intranet. To cover these challenges of deep learning tasks, we analyzed available options within current technologies. To the best of our knowledge, karas on the top of TensorFlow offers readily available models that offer classification features for 10,000 generic classes. Among the available options, we found three most-suitable pre-trained models that are smaller in size, lighter in computation for training, and faster in inference (see Table 2). 

Here,

- Parameters: These are the internal variables that the model adjusts during training to make accurate predictions.
- Total and Trainable: “Total” refers to the complete number of parameters in the model. “Trainable” specifies the subset of these parameters that are updated during training. Some parameters may be “frozen” to preserve certain functionalities.
- Inference Step: This refers to the process of using the trained model to make a prediction. The time taken for one inference step is critical in real-world applications, especially when computational resources are limited.

**Figure 2:**
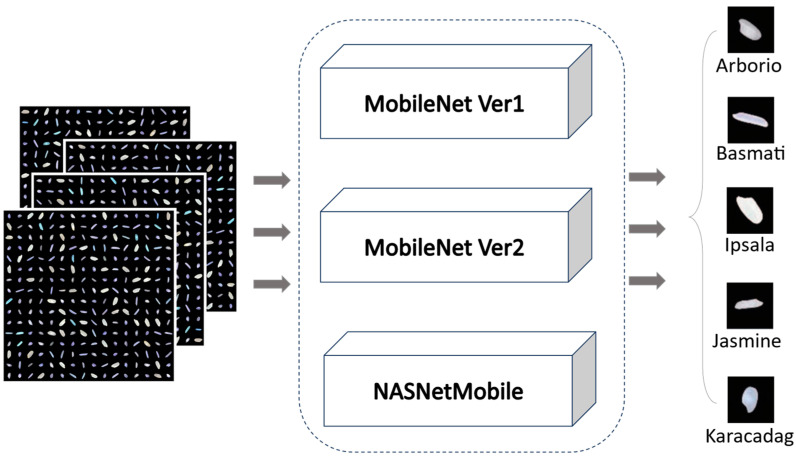
Proposed work to train the model end-to-end using lightweight architectures.

End-to-end learning has proven successful in complex domains like emotion recognition and Food Nutrition Estimation [35,36] -like studies. This motivates us to consider it for rice variety classification in this study. The proposed end-to-end deep learning classification of rice varieties uses lightweight models (see Figure 2). The dataset consists of five types of rice images each containing approx. 15,000 images. Our goal is to minimize the process of model training while maintaining the best performance with minimum model size so that it can be used in constrained-resource devices. After evaluating several models, we selected MobileNet v1 [37], MobileNet v2 [38], and NASNetMobile [39] models with imagenet’s [40] pretrained weights. Using a transfer learning strategy, few modifications were made in the last layers. Our results for classification accuracy, AuC, and Recall matrices are equal to and above the current benchmarks. However, the process has been optimized by eliminating pre-processing and handcrafted feature extraction. Moreover, we offer models that are smaller in size that makes them better options for mobile devices.

Andrew Howard and Mark Sandler et al. from Google proposed MobileNet’s first two versions [37,38] in 2017 and 2019, respectively. Meanwhile, NASNet’s mobile version was also proposed [39] from Google in 2018 by Barret Zoph et al. The common purpose of these models was to offer lightweight yet best performance for vision tasks. They are readily available in Tensorflow and can be used via a Keras wrapper with imagenet weights.

#### 3.2.1. MobileNets

**Figure 3:**
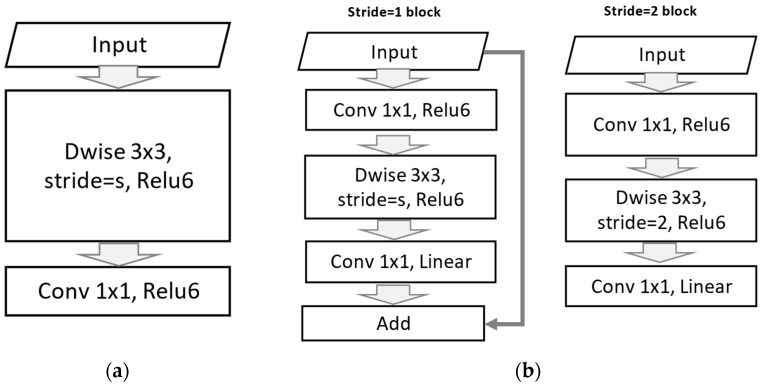
MobileNet convolution blocks. (**a**) v1 and (**b**) v2.

The first version employed Depthwise Separable Convolution to minimize the size and complexity of the model. This resulted in fewer parameters during training and lesser computations at inference. The two parameters termed Width Multiplier and Resolution Multiplier were introduced to make tuning easy. As can be seen in Figure 3a, the first layer of v1 (depthwise convolution) performs lightweight filtering using a single convolutional kernel per input channel. The second layer, know as pointwise convolution (1 × 1 convolution), is used to build new features by computing linear combinations from the input channels. ReLU6 has been used for comparison with low precision. 

In the second version, inverted residual structure has been introduced. This offers expansion-filtering compression by adding an expansion layer. It also removed nonlinearity in narrow layers. In Figure 3b, two block types can be observed, i.e., residual (stride = 1) and downsizing (stride = 2). Each block type consists of 3 layers. The first layer is 1 × 1 convolution, the second is depthwise convolution, and the last one is 1 × 1 convolution with linearity. 

#### 3.2.2. NASNetMobile

**Figure 4:**
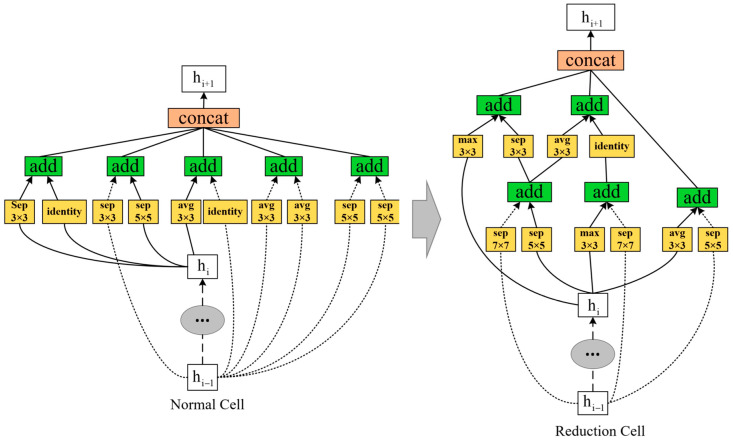
NASNetMobile: derived from Normal Cell to Reduced Cell.

Neural Architecture Search (NAS) refers to the technique for searching through a neural network’s space configurations. It formulates the task of finding the best-suited CNN architecture as a Reinforcement learning problem. At its core, the aim was to find the best combination of hyperparameters by rewarding accuracy at given datasets (CIFAR10 and ImageNet). The reduced form is called NASNetMobile (see Figure 4). 

### 3.3. Experimental Setup

#### 3.3.1. Dataset

**Figure 5:**
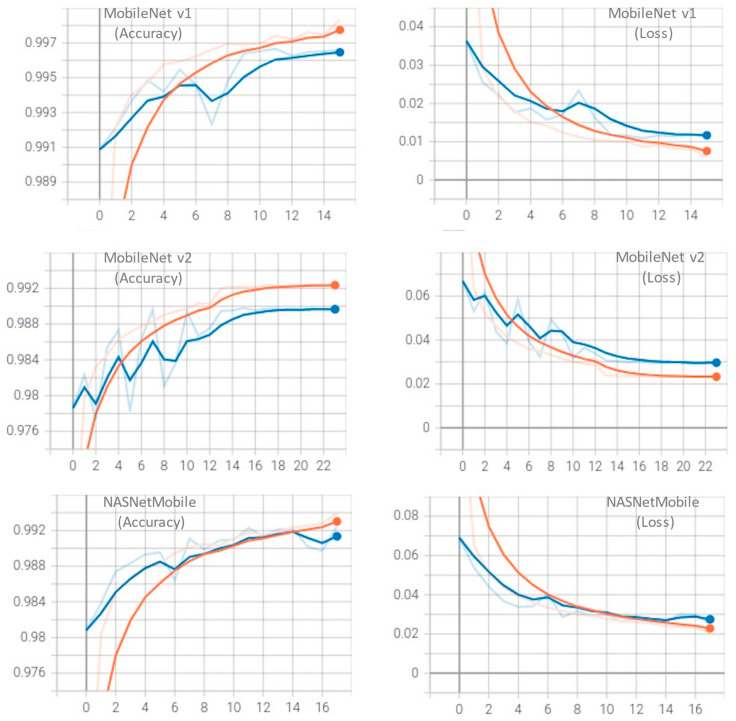
Training performance for classification accuracy. Orange curves refer to training and blue refer to validation performance.

The first step in training the image classifier was to organize the dataset into a root directory with five sub-directories, each containing images from a given class. The dataset obtained from Kaggle was already structured in this manner, so the next step was to split it into training and validation sets. The total number of images in the dataset was 60,000, which was split into an 80/20 ratio for the training and validation sets. Each class was equally divided into 12,000 images for the training set and 3000 images for the validation set (Figure 5). This was done to ensure that the model had a balanced and diverse set of images for training and a separate set of images for validation, which would be used to assess the performance of the model. The split between the training and validation sets was performed randomly to avoid any bias in the results.

The organized dataset and the split into the training and validation sets were crucial steps in training the image classifier as they helped to ensure that the model was trained on a diverse and representative set of images, and that its performance was accurately evaluated using a separate set of images. The 80/20 ratio between the training and validation sets was chosen to provide a large enough training set to train the model effectively, while still having a sufficiently large validation set to evaluate the model’s performance.

#### 3.3.2. Training 

The training performance of each model has been monitored using accuracy and loss matrices, which are displayed in Figure 5. These matrices provide a visual representation of how well the models were trained, and how they were able to improve over time. As illustrated, the MobileNet V1 model achieved its maximum performance at the 15th epoch, MobileNet V2 at the 23rd epoch, and NASNetMobile reached its maximum performance at the 18th epoch. The models were trained using the initial imagenet weights, and the batch size was set to 64, with a maximum of 50 epochs. An early-stopping strategy was employed to prevent overfitting, ensuring that the models were only trained for the necessary number of epochs.

In addition to these configurations, we also utilized keras callbacks to add additional functionality to the training process. These callbacks allowed for greater control over the training process, allowing the authors to fine-tune the models and achieve optimal performance. By using these techniques, we were able to train the models effectively and achieve high accuracy. These callbacks and hyperparameters have been summarized in Table 3.

1. TerminateOnNaN: To stop the training process when NaN (Not a Number) values are encountered in the training process. In deep learning models, NaN values can indicate that the model has encountered an issue during training that cannot be resolved. It helps to ensure that the models are trained on clean and reliable data, ensuring that the models are able to achieve optimal performance.
2. EarlyStopping (monitor = ‘val_loss’, min_delta = 0.001, patience = 6): To prevent overfitting when the model becomes too specialized for the training data and its performance on unseen data decreases. In our experiment, the criterion for EarlyStopping is based on validation loss. It waits for 6 epochs to see if a 0.001 improvement (decrease) has been made, otherwise it stops the training.
3. ReduceLROnPlateau (monitor = ‘val_loss’, factor = 0.01, patience = 5, mode = ‘auto’, min_delta = 0.001): To reduce the learning rate of the model during training which determines the size of the updates to the model’s weights during training. In general, a high learning rate can cause the model to oscillate and converge slowly, while a low learning rate can cause the model to converge too slowly. This callback helps to mitigate this issue by dynamically adjusting the learning rate during training, based on the performance of the model on the validation set. The callback monitors the validation loss, and if the loss does not improve for a certain number of epochs, the learning rate is reduced.

The training environment used in this study was a 64-bit Ubuntu 20.04.5 LTS operating system running on an Intel^®^ Core i5-3470 CPU @ 3.20GHz x 4 processor. The training was performed using an NVIDIA GeForce GTX 1080 GPU, which provided the necessary computing power to train the deep learning models effectively. The programming scripts for the training were written using Python 3.9.12 and utilized libraries such as tensorflow 2.4.1 and keras-gpu 2.4.3. These libraries provide a comprehensive set of tools for building and training deep learning models, making it easier for researchers to experiment with different models and parameters. The use of an optimized training environment and well-established libraries allowed for efficient and accurate training of the deep learning models, ensuring that the results of this study were robust and reliable. Furthermore, the use of Python and open-source libraries makes it easier for other researchers to reproduce and build upon this work.

#### 3.3.3. Pre-Processing and Data Augmentation

The hallmark of this study is the implementation of an end-to-end training framework that requires no manual feature selection or engineering, thereby significantly reducing the need for expert intervention. Despite this automated approach, specific pre-processing and data augmentation steps were integrated to ensure optimal model performance and generalizability.

Pre-processing Steps: In keeping with the end-to-end training philosophy, our pre-processing steps were minimal yet effective. All images were converted to grayscale to lessen the computational load. Additionally, pixel values were normalized to fall within a range from 0 to 1, facilitating better convergence during the training phase. All images were resized to a uniform dimension of 224 × 224 pixels to maintain consistency.

Data Augmentation Steps: To further bolster the model’s robustness within the end-to-end framework, we employed several data-augmentation techniques. These included rotation at varying angles (0, 90, 180, and 270 degrees), horizontal and vertical flipping, and random zoom within a pre-specified range. These steps were automated and integrated into the training pipeline.

The automated nature of these steps, in conjunction with our end-to-end training approach, highlights the paper’s significant contribution towards reducing both computational overhead and the dependency on specialized expertise.

#### 3.3.4. Assessing Model Complexity and Parameter Uncertainty

The complexity of the models used in this research—MobileNet v1, MobileNet v2, and NASNetMobile—is detailed in terms of the size, number of parameters, and time taken per inference step in Table 2. Despite the lightweight nature of these models, it is worth discussing the computational resources they consume:

- MobileNet v1: With 3233 total parameters, it remains computationally efficient, particularly for CPU-based inference.
- MobileNet v2: Slightly fewer parameters (2264) but comparable inference times make it another viable option.
- NASNetMobile: The most parameter-heavy among the three (4275), yet it offers a competitive inference time especially on a GPU.

Given that the models are pre-trained on ImageNet and fine-tuned for rice variety classification, there exists a certain level of parameter uncertainty. To mitigate this, we employed techniques like early stopping and monitored the performance matrices closely. While early stopping helps in preventing the model from overfitting, it also serves as a measure against parameter uncertainty by halting the training once no significant improvement is observed.

## 4. Results and Discussion

In this study, the classification of rice varieties using lightweight deep learning models has been investigated. This section discusses key findings and results in subsections. 

### 4.1. Why to Prefer CNN over DNN

One key advantage of CNNs over Densely Connected Neural Networks (DNNs) is their ability to effectively capture spatial features within an image. CNNs use convolutional layers that scan the input image and extract low-level features, which are then passed through additional layers that gradually learn more complex and abstract representations of the image. By doing this, CNNs are able to exploit the spatial relationships between pixels in an image, which is critical for image classification. In contrast, DNNs are typically composed of fully connected layers that treat the input as a one-dimensional vector, which means they are not optimized for processing images. The absence of convolutional layers in DNNs makes it difficult for them to capture the spatial dependencies between pixels and extract the features that are necessary for image classification. Furthermore, the high number of parameters required by DNNs to model image data can result in overfitting and slower training times compared to CNNs. In contrast, CNNs use parameter sharing to reduce the number of learnable parameters, which makes them more efficient and effective for image-classification tasks. Therefore, CNNs are the preferred choice for image-classification tasks due to their ability to effectively capture spatial features within an image, exploit spatial relationships between pixels, and reduce the number of learnable parameters. These properties make CNNs a highly effective and efficient solution for image-classification tasks compared to DNNs.

### 4.2. MobileNet Efficiency

MobileNet v1 utilizes depthwise separable convolutions, which factorize standard convolutions into two separate operations: a depthwise convolution and a pointwise convolution. This approach reduces the computational cost of the convolutional layers by a factor of the input channel size. It also utilizes a linear bottleneck function to further reduce the model size. MobileNet v2 utilizes an inverted residual structure that includes a shortcut connection between the input and output of each block. This structure allows for better gradient flow and faster training times. Its linear bottleneck function also contributes to an improvement in the representational capacity of the network. The performance of MobileNet v1 and v2 has been evaluated on the dataset for classification tasks. Both models have consistently demonstrated high accuracy and low model size compared to other state-of-the-art models. MobileNet v2 in particular has achieved new state-of-the-art performance on ImageNet with a model size of only 14 MB.

### 4.3. NASNetMobile Equalence to MobileNets

The performance of NASNetMobile has also been found to be equally as good as that of MobileNets. This study compared the performance of NASNetMobile to that of MobileNets and VGG nets on the given dataset. The results showed that NASNetMobile achieved equivalent accuracy to that of MobileNets while maintaining a similar model size. The effectiveness of NASNetMobile is attributed to the automated design process used to create the architecture. NAS allows for the exploration of a vast search space of possible architectures, which allows for the discovery of novel and effective network architectures. In addition, NASNetMobile utilizes a combination of depthwise separable convolutions and regular convolutions, which allows for a balance between model size and accuracy. 

### 4.4. Comparison with Previous Work

While previous studies have reported exceptional performance for CNNs [21], they have also been noted as computationally expensive. This makes them less feasible for resource-constrained devices or environments. To address this issue, the use of lighter models that offer similar performance to CNNs but with lower computational cost is proposed. It was demonstrated by our results that these lightweight models achieved comparable accuracy with a significantly lower number of parameters and computations, making them more suitable for real-world applications with limited resources.

This study establishes that both CNNs and lightweight models can proficiently classify rice varieties using deep learning. Nevertheless, the experiments reveal a trade-off between accuracy and computational cost. CNNs demanded substantially more computational resources, but they surpassed the lightweight models in terms of accuracy. This balance between performance and computational cost becomes a crucial factor when deploying deep learning models in practical scenarios. In some instances, the use of CNNs may be warranted, such as when achieving high accuracy is of utmost importance. On the other hand, lightweight models could serve as a more pragmatic solution in situations where computational resources are scarce. Overall, this study contributes to the ongoing pursuit of optimizing deep learning models for environments constrained by resources. The findings presented here are expected to inform the development of more efficient and effective deep learning models suitable for real-world applications. By identifying the trade-offs and potential benefits of both CNNs and lightweight models, this research offers valuable insights that can guide the selection and implementation of deep learning models in various settings, including those with limited computational resources.

The listed models achieve more than 99% performance on classification accuracy, precision, recall, and area under curve. 

**Figure 6:**
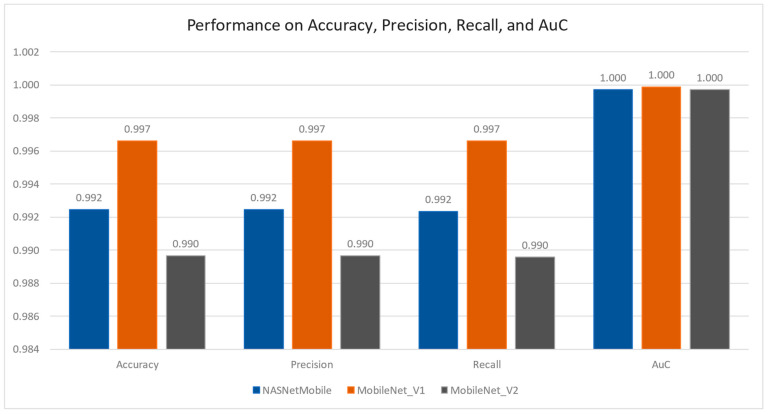
Classification Accuracy, Precision, Recall, and AuC for the trained models.

The results illustrated in Figure 6 and Table 4 show almost similar performance to those reported in [21], i.e., 99.87%, 99.95%, and 100% for ANN, DNN, and CNN. They have narrated their performance on classification accuracy while other matrices have not been calculated. 

The results illustrated in Figure 6 and Table 2 show almost similar performance to those reported in [21], i.e., 99.87%, 99.95%, and 100% for ANN, DNN, and CNN. They have narrated their performance on classification accuracy while other matrices have not been calculated. 

Moreover, from the CNN family, they used VGG-16 which comprised 16 layers but 138.4 million parameters (see Table 5). This makes the VGG-16 model 32 times more computationally expensive than MobileNet v1 and 39 times more than MobileNet v2. A similar baseline can also be found in [41] for rice classification using other datasets. Other than VGG-16, they have also employed ResNet variants which are also at least 5 times more computationally complex than our selected models. 

### 4.5. Generalization and Application

The robustness of our model was evident in its performance across a diverse set of rice varieties, laying the foundation for potential generalization to other rice varieties and even other grains. Importantly, our architecture is amenable to fine-tuning with additional datasets for new rice varieties as they become relevant in agricultural practices. This flexibility underscores the adaptability of our model, poised not merely as a static solution but as a dynamic tool capable of evolving alongside advancements in rice variety identification.

Mobile deployment signifies a transformative step in making our model practically applicable in real-world agricultural settings. Utilizing TensorFlow’s well-documented procedures for mobile implementation, the transition from a research prototype to an operational mobile application becomes a streamlined process. Additionally, architectures like NASNetMobile, MobileNet_V1, and MobileNet_V2 offer the necessary computational efficiency for mobile deployment without compromising on the model’s performance. This confluence of factors contributes to a balanced approach, optimizing both technological sophistication and practical utility.

While the proposed lightweight deep learning models significantly reduce computational requirements compared to traditional architectures, they are not entirely devoid of challenges. One of the main difficulties in applying the proposed method lies in the trade-off between performance and computational efficiency. Despite their lighter footprint, these models may still require fine-tuning and optimization to achieve the desired level of accuracy, particularly when dealing with diverse and complex rice varieties. This fine-tuning can introduce additional layers of complexity, potentially requiring expertise in machine learning and computational resources that may not be readily available in all settings.

### 4.6. Performance–Efficiency Trade-Off in Lightweight Models

The findings of this study underscore the potential of lightweight deep learning models in rice variety classification, particularly in resource-constrained environments. However, it is crucial to consider the inherent trade-offs between computational efficiency and model performance. While the proposed models demonstrate a reduction in computational requirements, achieving the desired level of accuracy may necessitate fine-tuning, thereby introducing additional computational and expertise-related challenges. This brings forth an essential question: Can lightweight models ever match the performance of their more resource-intensive counterparts without significant trade-offs?

Furthermore, this study opens up avenues for exploring hybrid models that could potentially combine the advantages of both deep learning and classical machine learning techniques. Such hybrid models could offer a balanced compromise between computational efficiency and classification accuracy, a topic worth exploring in future research.

## 5. Conclusions

This paper focuses on using lightweight deep learning models for the classification of rice varieties and discusses the potential of these models in edge computing scenarios. Specifically, the methodologies employed include lightweight convolutional neural networks (CNNs) and MobileNet. These models are selected for their computational efficiency, making them suitable for deployment in edge computing devices. The presented methodology harnesses deep learning capabilities for vision tasks in agricultural fieldwork. The approach is demonstrated using a rice image dataset, showing that compact models can achieve precise image classification even in offline mode. Designed as an end-to-end solution with minimal human expert intervention, this methodology offers an effective and efficient option for the agricultural domain. The findings of this study underscore the potential of deep learning in enhancing the accuracy and efficiency of vision tasks in agricultural fieldwork. The employment of small-sized models, such as MobileNet v1 and v2 and NASNetMobile, confirmed that these models can deliver accurate results without relying on high-speed internet connectivity, which is particularly beneficial in agricultural settings where such access may be limited. This research contributes to the application of deep learning in agricultural fieldwork and opens up possibilities for further advancements in this area. Future work will seek to expand upon this foundation by addressing additional vision tasks, such as plant disease detection and segmentation, further enriching the potential applications of deep learning in agriculture.

While this paper provides a comprehensive approach to rice variety classification using lightweight models, there are areas that could benefit from further research:

- Scalability: As the number of rice varieties increases, the model needs to scale effectively to maintain high classification accuracy.
- Real-world Validation: This paper could elaborate on how the model performs in various real-world conditions, such as different lighting and backgrounds, which are critical for practical deployment.
- Generalizability: Although this paper does discuss potential generalizability, it could benefit from empirical studies to confirm how well the model adapts to new rice varieties or even other types of grains.

## Figures and Tables

**Table 1:** Summary of focused research findings, challenges, and contributions.

Area	Existing Research and Findings	Identified Challenges	Contribution of This Study
Deep Learning in Agricultural Applications	Deep learning has shown promise in improving crop yields and resource optimization [20].	High computational cost and complexity, especially for deep architectures.	Introduces lightweight models that maintain performance but reduce computational complexity.
Case Studies and Custom Models	Studies like [8,9] focus on specific applications like fruit counting and crop classification. Lighter models for disease detection are discussed in [11].	These models often require expert-level feature engineering and pre-processing.	Aims to reduce dependency on expert-level pre-processing and feature engineering.
Performance Metrics in Existing Models	High performance reported for ANN, DNN, and CNN models in studies like [21,22].	Focus primarily on performance, overlooking computational cost and pre-processing requirements.	Balance between performance and computational efficiency, reducing the need for expert intervention.

**Table 2:** Top 3 models with optimal statistics for resource-constrained environments.

Model	Size (MB)	Parameters (k)		Time (ms) per Inference Step	
		Total	Trainable	CPU	GPU
MobileNet v1	16	3233	5	22.6	3.8
MobileNet v2	14	2264	6	25.9	3.4
NASNetMobile	23	4275	5	27	6.7

**Table 3:** Summary of hyperparameters used in training.

Parameter/Setting	MobileNet V1	MobileNet V2	NASNetMobile	Description
**Epoch for Max** **Performance**	15th	23rd	18th	Epoch at which the model achieved its maximum performance
**Batch Size**	64	64	64	Number of samples processed before updating the model weights
**Max Epochs**	50	50	50	Maximum number of epochs for training
**Early Stopping**	Yes	Yes	Yes	To prevent overfitting, based on **val_loss, min_delta = 0.001, patience = 6**
**Terminate On NaN**	Yes	Yes	Yes	Stops training if NaN values are encountered
**ReduceLR On** **Plateau**	Yes	Yes	Yes	Reduces learning rate if **val_loss** does not improve, **factor = 0.01, patience = 5, min_delta = 0.001**
**Initial Weights**	Imagenet			Pre-trained weights used for initialization
**Training** **Environment**	Ubuntu 20.04.5 LTS, Intel Core i5-3470 CPU, NVIDIA GeForce GTX 1080, Python 3.9.12, tensorflow 2.4.1, keras-gpu 2.4.3			Software and hardware environment used for training

**Table 4:** Results of Classification Accuracy, Precision, Recall, and AuC for the trained models.

Model	Accuracy	Precision	Recall	AuC
NASNetMobile	99.2	99.2	99.2	100.0
MobileNet_V1	99.7	99.7	99.7	100.0
MobileNet_V2	99.0	99.0	99.0	100.0

**Table 5:** Computational cost of models with respect to their parameters.

Proposed	Model	Parameters	Depth
Our Proposal	NASNetMobile	**5.3 M**	99.2
	MobileNet_V1	**4.3 M**	55
	MobileNet_V2	**3.5 M**	105
Koklu et al. [21]	VGG-16	138.4 M	16
Chatnuntawech [34]	ResNet-B	35.76 M	116

## Data Availability

The data used to support the findings of this study can be made available by the corresponding author upon request.
